# White Matter Fiber Bundle Alterations Correlate with Gait and Cognitive Impairments in Parkinson’s Disease based on HARDI Data

**DOI:** 10.2174/0115734056330364250109072154

**Published:** 2025-01-14

**Authors:** Lining Dong, Mingkai Zhang, Zheng Wang, Ying Yan, Ran An, Zhenchang Wang, Xuan Wei

**Affiliations:** 1 Department of Radiology, Beijing Friendship Hospital, Capital Medical University, No. 95, Yong An Road, Xicheng District, Beijing 100050, China; 2 Department of Neurology, Beijing Friendship Hospital, Capital Medical University, Beijing 100050, China; 3 Department of Neurology, Xuanwu Hospital of Capital Medical University, Beijing 100053, China

**Keywords:** Parkinson’s disease, Diffusion magnetic resonance imaging, High angular resolution diffusion imaging, Cingulum cingulate, Gait impairment

## Abstract

**Background:**

The neuroanatomical basis of white matter fiber tracts in gait impairments in individuals suffering from Parkinson’s Disease (PD) is unclear.

**Methods:**

Twenty-four individuals living with PD and 29 Healthy Controls (HCs) were included. For each participant, two-shell High Angular Resolution Diffusion Imaging (HARDI) and high-resolution 3D structural images were acquired using the 3T MRI. Diffusion-weighted data preprocessing was performed using the orientation distribution function to trace the main fiber tracts in PD individuals. Clinical characteristics between the two groups were compared, and the correlation between the FA value and behavioral data was analyzed. Quantitative gait and clinical parameters were recorded in PD at ON and OFF states, respectively.

**Results:**

The mean tract-specific FA values of the right Cingulum Cingulate (rCC) were statistically different between the PD group and the HC group (*p* =0.047). The FA value of 34-58 equidistant nodes in rCC was positively correlated with Mini-Mental State Examination (MMSE) (r=0.527, *p*=0.024), Berg Balance Scale (BBS)-OFF (r=0.480, *p* =0.040), and BBS-ON (r=0.528, *p* =0.024) scores, while it was negatively correlated with the MDS-UPDRS-III-ON score (r=-0.502, *p* =0.030). Regarding the gait analysis, the FA value was significantly correlated with velocity, cadence, and stride time of the pace and rhythm domains in both ‘ON’ and ‘OFF’ states, respectively (*p*<0.05).

**Conclusion:**

This study served as an initial exploration to establish that HARDI sequences could be employed as a robust tool for analyzing microstructural alterations in white matter fiber bundles among PD patients, although the sample size was small. We confirmed microstructural integrity impairment of rCC to be significantly associated with both gait and cognitive deficits in patients with PD. Early detection of microstructural changes in rCC and targeted treatment can help improve behavioral disorders. In the future, we intend to further integrate multimodal data with assessments of patient behavior both prior to and following intervention. We will validate our findings within an independent cohort to monitor disease progression and evaluate the efficacy of therapeutic interventions.

## 1 INTRODUCTION

Parkinson's Disease (PD) is a common neurodegenerative disease, second only to Alzheimer's [1]. The main clinical manifestations are motor symptoms and non-motor symptoms that progressively get worse, and have an impact on function and quality of life [2]. The typic motor symptoms include akinesia/bradykinesia, rigidity, tremor, postural instability, and motor initiation disorder [*e.g*., Freezing of Gait (FOG)] [3, 4].

Gait impairments occur early, especially in those who have Postural Instability and Gait Disturbances (PIGD) phenotype. Reduced stride, steady-state decline, reduced walking speed, and other symptoms are common [5]. With disease pro-gression, gait and balance problems are further aggravated, and the patients may fall [6]. In addition, anxiety and fear of falling resulting from gait impairments are common in individuals suffering from PD [7]. Gait can be modelled into 5 domains: pace, rhythm, variability, asymmetry, and postural control [8]. We can conduct objective gait evaluations using a 20-foot-long computerized Zeno Walkway system (Proto Kinetics, Havertown, PA, USA) at a Self-selected Pace (SSP) and a Fast Pace (FP) [9].

The pathology of PD has an influence on many brain regions when the disease progresses [10, 11]. For example, besides major basal ganglia and substantia nigra (SN) pars compacta (SNpc), the cortex, thalamus, and other subcortical structures are affected as well [12]. These brain regions are directly and indirectly linked to gait impairments. A previous study has found altered vermal functional connectivity with bilateral paracentral lobules to be correlated with measures of variability in stride length, step time, and single support time [13]. Additionally, in the ON state, alterations in the connectivity between the right pedunculopontine nucleus and the caudate nucleus are associated with deterioration in cadence, stride time, and velocity [14]. Similar observations have been noted within the thalamus [15]. Our previous study found the surface area changes of the left Lateral Temporal Cortex (LTC) and right Inferior Parietal Cortex (IPC) to be associated with the pace domain of the gait in the ON state [9]. Dysfunction in each gait domain may correlate with abnormal changes in specific areas [16].

Some studies have confirmed that the degenerative changes of PD can lead to the loss of neurons and the damage of fiber tracts [17]. Utilizing Diffusion-weighted Imaging (DWI) data, tractography offers a non-invasive approach to identifying the primary white matter pathways within the brain [18]. The Diffusion Tensor Imaging (DTI) model is the most commonly used approach based on DWI data [15, 19]. However, the main disadvantage is that it cannot restore more than one fiber orientation in a single voxel. Another model, known as High Angular Resolution Diffusion Imaging (HARDI), can overcome the limitation of DTI. This approach reveals more intricate structures, such as the crossover and bifurcation of fiber tracts. Thus, it provides more accurate fiber tracts [20] with higher resolution with regard to fiber tract direction and connection [21]. HARDI can also segmentally display the local fiber tracts, which also have a qualitative leap in accuracy compared to DTI [22]. As a result, more subtle microstructure changes can be detected [23, 24].

Fractional Anisotropy (FA) represents a scalar measure-ment of diffusion for HARDI, and an indirect proxy for the white-matter tract microstructure [25, 26]. Neuronal degeneration eliminates diffusion barriers and their orientation dependence, leading to a decreased FA, which was found in the PD-related SNpc [27]. Similar diffusion changes were observed in the thalamus [28], caudate [15, 19], putamen [29], globus pallidus [19], and brainstem structures, including the pedunculopontine nucleus [15, 30, 31]. HARDI may help characterize fiber loss in diseases associated with white matter degeneration [32]. HARDI data have been used in several PD studies. For example, in the Parkinson's Progression Markers Initiative (PPMI) dataset, compared to Healthy Controls (HCs), generalized FA and maximal apparent fiber density in the pathways connecting SN in PD individuals were significantly increased, while the same metrics decreased in the fiber tracts connecting corpus callosum [33].

Spherical Deconvolution (SD) based on HARDI can estimate the full fiber Orientation Distribution Function (ODF) in each brain voxel. The Constrained SD (CSD) could decrease the noise sensitivity, thus enabling analyzing HARDI data easier [34], and achieve more accurate whole-brain fiber tracts [35]. In our study, based on HARDI data, we utilized the Multi-shell Multi-tissue CSD (MSMT-CSD) method to estimate ODF. The ODF was thereafter applied to trace the main fiber tracts in individuals suffering from PD. We supposed that alterations in the microstructure of the fiber tracts of HARDI could be associated with cognitive decline and gait disorders in PD patients. We subsequently utilized this index, the mean FA value (collectively referred to as FA), to measure the characteristics of the PD and analyzed the correlation between the microstructural changes of brain tissue and the multi-dimensional clinical and gait data in PD individuals.

## 2 METHODS

### 2.1 Participants Enrollment

Individuals bearing PD and receiving dopaminergic medications were recruited from the Movement Disorders Program at Beijing Friendship Hospital of the Capital Medical University. All PD participants were assessed by movement disorder neurologists who confirmed the diagnosis of idiopathic PD based on UK Parkinson’s Disease Society Brain Bank criteria [36]. The exclusion criteria and recruitment of HCs were consistent with our previous studies [37]. Twenty-four individuals living with PD and 29 HCs were enrolled. Table **1** shows demographics and clinical statuses. We evaluated the International Parkinson and Movement Disorder Society Unified Parkinson's Disease Rating Scale (MDS-UPDRS) part-III and MDS-UPDRS-total. For all participants, we employed Berg Balance Scale (BBS), the Mini-Mental State Examination (MMSE), the Beck Depression Inventory (BDI) score, the Beck Anxiety Inventory (BAI) score, Modified Apathy Evaluation Scale (MAES), Parkinson’s Disease Questionnaire-39 questions (FDQ-39), New Freezing of Gait Questionnaire (NFOGQ), and the Timed-Up and Go (TUG) tests. Objective gait assessments, including velocity, cadence, stride time, and stride length, at a Self-selected Pace (SSP) and a fast pace (FP), were also assessed. The above scales were consistent with our previous research [9].

### 2.2 MRI Data Acquisition and Preprocessing

Diffusion-weighted images were acquired using a 3.0 T Siemens Prisma scanner (Siemens, Munich, Germany) with an eight-channel head coil. For the data acquisition and preprocessing of DWI and 3D T1-weighted images, we referred to our previous study [38].

### 2.3 Whole Brain Tractography and Fiber Quantification

**Fig. (1):**
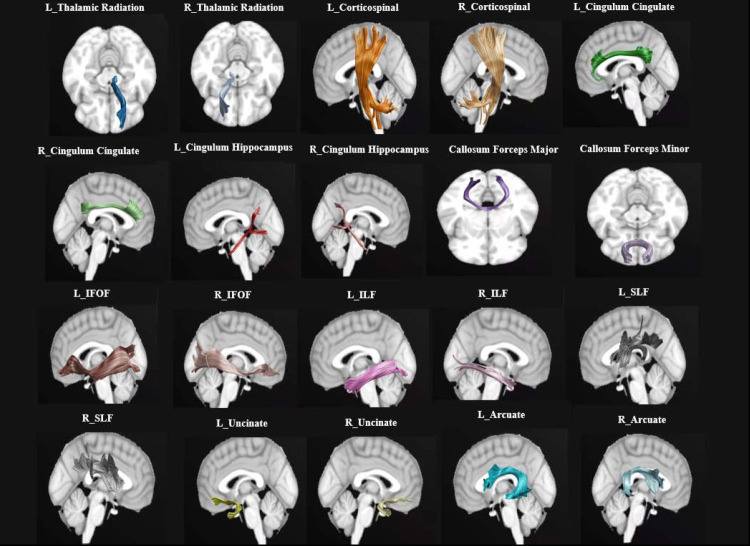
Twenty fiber tracts were investigated in the present study. The fiber tracts were extracted by automated fiber quantification.

For each subject, multi-shell multi-tissue response functions were computed with the Dhollander algorithm using the *dwi2 response* function in MRtrix3. MSMT-CSD method was then used to estimate multi-tissue ODF [35]. Whole-brain streamline tractography was performed using the SD_ STREAM method and the function of *tckgen* in MRtrix3. The main tracking parameters included the following: desired number of streamlines = 1000000, step size = 0.5 x voxel size, angle = 45°. Additionally, DWI and FA maps were calculated with the *dtifit* function in FSL [39]. Next, we performed Automated Fiber Quantification (AFQ) analysis (version 1.2; Stanford University, California, USA) [18] to extract the 20 major white matter tracts and segment each tract into 100 equidistant nodes in each participant. The AFQ analysis included the following primary steps: [1] loading the whole-brain tractography result obtained from MRtrix3 into AFQ software; [2] extracting the 20 tracts from the whole-brain tracts by two waypoint regions-of-interest included in AFQ software; [3] refinement of the fiber tracts, which was accomplished by comparing each candidate fiber to fiber tract probability maps; [4] removing fibers that are more than 4 standard deviations above the mean fiber length or that deviate more than 5 standard deviations from the core of the fiber tract iteratively to get a compact bundle for each tract; [5] segmentation of each tract into 100 equidistant nodes along the central portion of the bundle, and calculation of the FA value of each node. The 20 white matter tracts included left/right thalamic radiation, left/right corticospinal tract, left/right cingulum cingulate, left/right cingulum hippocampus, callosum forceps major/minor, left/right inferior fronto-occipital fasciculus, left/right inferior longitudinal fasciculus, left/right superior longitudinal fasciculus, left/right uncinate, and left/right arcuate (Fig. **1**).

### 2.4 Statistical Analysis

Demographic and clinical characteristics were described as percentages for categorical variables and median with interquartile ranges for continuous variables. Comparisons between PD individuals and HCs were performed based on the chi-square test for categorical variables and the Wilcoxon rank sum test for continuous variables. Spearman's correlation was calculated to assess the relationship between the mean FA value and patient-related rating scale (MDS-UPDRS, BAI, BDI, MMSE, MAES, FDQ-39, NFOGQ, TUG, and BBS) scores. In each analysis, age, gender, and education level were adjusted as covariates.

Objective gait parameters of PD and healthy individuals were compared using the Wilcoxon rank sum test. For PD subjects, changes between the ON and OFF states were analyzed using a paired two-sample Wilcoxon rank sum test. The correlation between the FA values and gait parameters was analyzed using Pearson's correlation analysis in the PD group. All analyses were performed using SPSS version 22.0 (version 22.0, IBM Comp. & SPSS Inc., 2010).

The differences in mean FA value between the HC and PD groups in the 20 major white matter tracts were compared based on the general linear model. The threshold for statistical significance was set at *p* < 0.05. Furthermore, more accurate node-wise comparisons were acquired to investigate the variation along the tract trajectory with significant differences. The FA values of 100 nodes were fed into permutation-based statistical analysis with 10000 permutations using the FSL Randomize program, with age as a covariate. The statistical results were subject to Family-wise Error (FWE) corrections for multiple comparisons following Threshold-free Cluster Enhancement (TFCE) and *p* value < 0.05 was considered significant.

## 3 RESULTS

### 3.1 Demographics and Behavioral Measurements of the Participants

This study included 24 patients and 29 HCs. Demographic and clinical data are shown in Table **1**, and objective gait analysis is shown in Table **2**. Testing for normality indicated that the normality assumptions were not met by all clinical and objective gait data (Tables **1** and **2**). There was no difference regarding the age, gender, education, MMSE, and MAES scores between the two groups. BAI, BDI, and TUG scores were significantly higher in the PD group than those in the control group (Table **1**), and the BBS score was significantly lower in the PD group, implying more anxiety/depression issues and worse gait and balance in PD participants. In terms of objective gait testing, PD subjects showed lower velocity and decreased stride length when compared to the HCs whether at SSP or FP (Table **2**). The velocity and stride length at FP were improved from the OFF state to the ON state, indicating responsiveness to dopaminergic treatment (Table **2**).

### 3.2 Group Differences in White Matter Average and Node-wise Levels

**Fig. (2):**
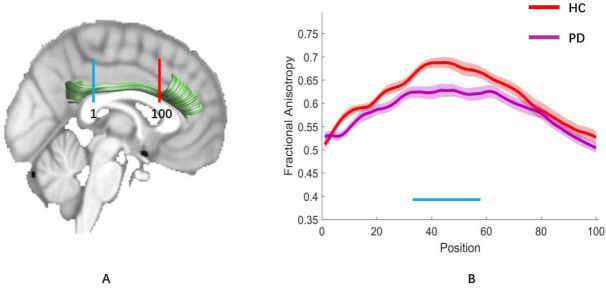
Nodewise comparisons for fractional anisotropy measurements along the rCC between healthy controls and patients. (**A**) rCC extracted by automated fiber quantification. The blue line represents the starting ROI, and the red line represents the ending ROI. (**B**) Nodewise comparisons along the rCC. Red indicates healthy control individuals, and purple indicates patients in mean (standard deviation) (solid lines represent mean and shaded areas indicate standard deviations). The horizontal scale represents 100 equidistant nodes along the central portion of the tract, defined by the blue starting point and red ending point (**A**). The blue bar under the FA profile indicates the nodes of significant difference between the PD and HC groups. A threshold of *p* < 0.05 was corrected by Family-wise Error (FWE).

Based on an AFQ analysis of the HARDI data, we extracted the FA values of the entire fiber tracts and compared the differences between the groups. The mean tract-specific FA values of the right Cingulum Cingulate (rCC) of the PD group were statistically lower than those in the HC group (*p*=0.047) (Table **3**). With more accurate node-wise comparisons and the tract trajectory analysis, the FA values of 34-58 equidistant nodes in the rCC of the PD group were significantly lower than those in the HC group (Fig. **2**).

### 3.3 Relationship between rCC and Neurocognitive and Objective Gait Measurements

**Fig. (3):**
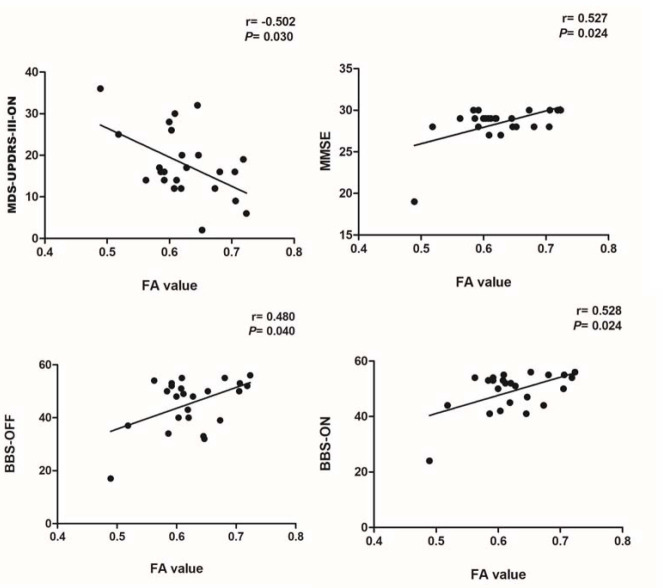
Correlation relationship between neurocognitive aspects and rCC in the PD group. The FA values of 34-58 equidistant nodes in the rCC were positively correlated with the MMSE, BBS-OFF, and BBS-ON scores, and negatively correlated with the MDS-UPDRS-III-ON score.

The FA values of 34-58 equidistant nodes in the rCC were positively correlated with the MMSE (r=0.527, *p*=0.024), BBS-OFF (r=0.480, *p*=0.040), and BBS-ON (r=0.528, *p*=0.024) scores (Fig. **3**), respectively. A negative correlation was detected with the MDS-UPDRS-III-ON score (r=-0.502, *p*=0.030) (Fig. **3**). Nevertheless, no significant correlation was identified among the BDI, BAI, MAES, PDQ-39, MDS-UPDRS-III-OFF, TUG, or NFOGQ scores.

**Fig. (4):**
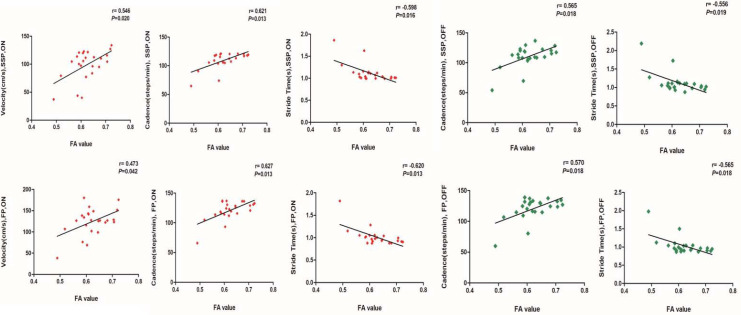
Correlation between rCC and gait assessments in both the ON and OFF states. FA stands for mean fractional anisotropy values of 34-58 equidistant nodes. In the ‘ON’ state (red), higher FA values of 34-58 equidistant nodes in the rCC were negatively correlated with the stride time SSP and stride time FP, and positively correlated with the velocity SSP, cadence SSP, velocity FP, and cadence FP. In the ‘OFF’ state (green), higher FA values of 34-58 equidistant nodes in the rCC were negatively correlated with the stride time SSP and stride time FP, and positively correlated with the cadence SSP and cadence FP.

Correlations were explored between FA values and gait assessments in both ON and OFF states. The FA values of 34-58 equidistant nodes in the rCC showed a stronger correlation with the pace and rhythm domains of the objective gait measurements (Fig. **4**). In the ON state, FA values of 34-58 equidistant nodes in the rCC were negatively correlated with the stride time at SSP (r=-0.598, *p*=0.016) and FP (r=-0.620, *p*=0.013), but positively correlated with the velocity at SSP (r=0.546, *p*=0.020), the cadence at SSP (r=0.621, *p* =0.013), the velocity at FP (r=0.473, *p*=0.042), and the cadence at FP (r=0.627, *p*=0.013). In the OFF state, FA values of 34-58 equidistant nodes in the rCC were negatively correlated with the stride time at SSP (r=-0.556, *p*=0.019) and FP (r=-0.565, *p*=0.018), while positively correlated with the cadence at SSP (r=0.565, *p*=0.018) and FP (r=0.570, *p*=0.018) (Fig. **4**).

## 4 DISCUSSION

The primary innovation of this study lies in its focus on an emerging topic: the neuroanatomical basis of gait impairments in Parkinson's Disease (PD), addressing the previously insuffi-cient evidence regarding the correlations between imaging findings and clinical gait parameters in PD. We explored the white matter fiber tracts of PD patients based on HARDI data. The main finding of the study is that there was a significant difference in mean FA values between the PD and HC groups in the right cingulate gyrus. In particular, the FA values of 34-58 equidistant nodes in the rCC were significantly lower in the PD group than in the HC group. In addition, the FA values of 34-58 equidistant nodes in the rCC were positively correlated with MMSE, BBS-OFF, and BBS-ON scores, and negatively correlated with the MDS-UPDRS-III-ON score. In the gait analysis, the FA value significantly correlated with velocity, cadence, and stride time of the pace and rhythm domains in both the ON and OFF states.

Neuronal degeneration removes the diffusion barrier and its orientation dependence, resulting in significantly lower FA values in the PD group than in the HC group. Our results showed a correlation between behavioral differences and FA values in rCC of individuals living with PD, rather than in the left cingulum cingulate. The role of spatial cognition [40] and action inhibition [41] is significantly influenced by the right hemisphere, with dysfunction in these areas potentially presenting as dyskinesia in Parkinson's disease. For example, functional topologies of spatial cognition predict motor progression, and cognitive impairment is associated with slow and short-stepped gait [42, 43]. Gait disorder in PD may involve a fundamental disruption to the brain's inhibitory control system [44]. A growing number of fuctional MRI studies suggest that the right hemisphere circuit appears to be more affected than the left hemisphere in individuals presenting FOG [45, 46]. Supportingly, Fling *et al*. [47] also reported that the individuals presenting FOG had significantly reduced volume of right pedunculopontine nucleus fiber tract when compared to HCs and those without FOG; FOG was proved to be strongly associated with structural deficits in the right motor brain networks. Tessitore *et al*. [46] reported reduced functional connectivity within the executive attention network of the right middle prefrontal gyrus in individuals presenting FOG. Collectively, these studies showed right brain structural and functional network connectivity to be altered in PD individuals, especially in gait parameters. Our results were similar to the above studies, with significant impacts on right fiber bundles in PD, and not the left.

The cingulate gyrus is mainly responsible for emotion, response selection, and feedback-guided decision-making, as well as visuospatial orientation and so on [48]. A previous study showed apathy in individuals living with PD to be manifested by decreased FA in the pregenual anterior cingulate cortex [49]. Unfortunately, our study failed to detect a correlation between FA values and emotional states. However, a positive correlation was found between FA values and MMSE, echoing the previously observed relationship between the integrity of the cingulum cingulate and cognitive function [50, 51]. Moreover, the balance ability of individuals living with PD may gradually decrease as part of the motor symptoms during the progression of the disease [52]. Our results showed the FA value of rCC to be positively correlated with the scores of BBS in ON and OFF states, while more severe cingulate injury was associated with poorer postural stability. The reason for this situation may be that the posterior mid-cingulate participates in the process of rapid body movement regulation in rCC [53], which affects body balance. The FA value was negatively correlated with the score of MDS-UPDRS-III-ON; precisely, the higher the score was, the more serious the motor symptoms were, indicating a significant correlation between the integrity of nerve fiber bundles in this segment of rCC and the severity of motor symptoms in individuals living with PD.

In terms of gait parameters, we found a correlation between the FA change in the 34-58 equidistant nodes of the rCC and the velocity, cadence, and stride time of PD subjects. The midcingulate cortex plays a crucial role in the regulation of skeletomotor control and orientation [54]. It has been suggested that the anterior part of the midcingulate cortex is described as anticipating and signalling motivationally relevant targets, encoding reward values, signalling errors, and influencing motor responses [54]. A Magnetoencephalogram (MEG) study based on individuals living with PD showed that the midcingulate participates in movement by integrating sensorimotor feedback [55]. The results of a resting functional Magnetic Resonance Imaging (rs-fMRI) study showed FOG, poor gait, and cognitive impairment in individuals living with PD to be significantly associated with reduced functional connectivity in large mesocortical networks containing the midcingulate [51]. In addition, studies have suggested that the corpus callosum and cingulum may lead to gait disorders and FOG by affecting gait planning ability [56, 57]. All the above studies have found the cingulate gyrus to play an important role in gait and showed that human gait control requires the integration of movement, perception, and cognition. A study showed that amyloid deposition in the cingulate gyrus can lead to worse walking conditions in the elderly population, including PD subjects [58]. Greater amyloid-beta deposition was associated with slower gait speed, lower cadence, and longer double support time [58]. Consequently, lower velocity and decreased stride length in PD may reflect a state characterized by a general decline in cingulate gyrus function. From OFF to ON state, we further noted that the FA values of 34-58 equidistant nodes in the rCC exhibited an increased correlation with stride time and cadence at both SSP and FP. A similar change has been found in the thalamus, putamen, and mesencephalic locomotor centres, but this phenomenon has never been investigated [15]. These findings may indirectly indicate a beneficial effect of dopamine therapy on rCC integrity, as correlation analyses suggest that rCC integrity is undergoing recovery during this period. These results could enhance our understanding of the neuropathological basis for gait impairment in individuals living with PD and its potential relationship with cognitive deficits.

The strengths of the study include that we applied the HARDI data for the first time to evaluate the relationship among objective gait parameters, behavioral scores, and the mean FA value. We also assessed clinical and gait data in detail by using computerized objective gait measurements in both ON and OFF states. Compared to the conventionally employed DTI model, HARDI offers enhanced accuracy and better microstructure detail [32]. Consequently, this study demonstrated the efficacy of HARDI in analyzing microstructural changes within white matter fiber bundles in patients with PD. Furthermore, our findings have revealed abnormalities in rCC. There are certain limitations in this study. The sample size of this study was small and it was a single-center study. Second, because of the sample size, differences in other fiber tracts were not found. The above aspects would have caused some of our results to be negative, but we still obtained some valuable findings. In future research, we will expand the sample size and further explore the differences in more fiber tracts. We can explore specific metrics in the fiber tracts, such as the Neurite Density Index (NDI) for determining the packing density of axons or dendrites, Orientation Dispersion Index (ODI) to assess the orientation coherence of neurites, and the Isotropic volume fraction (ISO) to quantify the fraction of isotropic freely diffusing water molecules within the voxel based on diffusion-weighted imaging data.

## CONCLUSION

Our findings have substantiated the existence of correlations between the rCC and the gait and cognitive processes. We have confirmed these impairments in the microstructural integrity of specific segmental fiber bundles to be significantly associated with both gait and cognitive deficits in patients with PD. Early detection of microstructural changes in rCC and targeted treatment can help improve behavioral disorders. Current treatment modalities do not consistently achieve optimal therapeutic outcomes for all patients. Consequently, there remains an urgent necessity for precise and effective treatment strategies addressing the motor disorders and cognitive impairment mechanisms associated with PD, particularly in relation to specific brain regions. This study has served as an initial exploration to establish that HARDI sequences can be employed as a robust tool for analyzing microstructural alterations in white matter fiber bundles among PD patients. HARDI can be combined with gait parameters to provide insights into the diagnosis and treatment of PD. In the future, we intend to further integrate multimodal data with assessments of patient behavior both prior to and following intervention. We will validate our findings within an independent cohort to monitor disease progression and evaluate the efficacy of therapeutic interventions.

## Figures and Tables

**Table 1:** Demographic and clinical characteristics of PD patients and healthy controls.

Measurements	Controls	PD	
		ON State	OFF State
Age	64 (51-70)	68 (63-70)	
Gender (male/female)	14/15	10/14	
Education (year)	10 (9-15)	10 (9-12)	
Duration of disease (month)	n/a	52 (27.3-81.8)	
LEDD (mg)	n/a	550 (318.8-743.8)	
MMSE	29 (27-30)	29 (26 -29.75)	
MAES	7 (5 -13)	10.5 (4.25-17)	
BAI	24 (22-25)	26 (24-29.75)**	
BDI	3 (1-4.50)	6 (4-9)**	
NFOGQ	n/a	10 (54.6)	
FDQ-39	n/a	20.50 (11.5- 33.3)	
MDS-UPDRS-III	n/a	16 (12.5-23.8)	29.50 (20-36.8)^##^
MDS-UPDRS Total	n/a	39.5 (25-46.8)	48 (34.3-62.5) ^##^
TUG	8.08 (7.28-8.63)	8.85 (8.16-10.8)**	10.4 (8.8-11.9)** ^##^
BBS	56 (54-56)	52 (44-54)**	48.5 (37.5-52.8)** ^##^

**Note: **PD, Parkinson’s disease; LEDD, levodopa equivalent daily dose (mg); MMSE, Mini-Mental State Examination; MAES, Modified Apathy Evaluation Scale; BAI, Beck Anxiety Inventory score; BDI, Beck Depression Inventory score; NFOGQ, New Freezing of Gait Questionnaire; PDQ-39, Parkinson’s disease Questionnaire-the 39 questions; MDS-UPDRS, International Parkinson and Movement Disorder Society-Unified Parkinson’s Disease Rating Scale; MDS-UPDRS-III, MDS-UPDRS motor score; TUG, Timed up and Go Test; BBS, Berg Balance Scale; n/a, not applicable.

For control, there is no ON or OFF state. Same set of clinical data from the control was used to analyze against PD group.

**, *p* < 0.01, compared with control group.

^##^, *p* < 0.01, compared with ON State.

**Table 2:** Objective gait parameters of the PD patients and healthy controls.

Characteristics	Controls	PD (ON State)	PD (OFF State)
Velocity SSP (cm/s)	116.96 (106.93-129.36)	105.16 (86.52-118.85)**	104.35 (79.08-113.49)**
Cadence SSP (step/min)	109.90 (106.82-119.79)	114.34 (105.54-119.13)	104.35 (79.08-113.49)
Stride time SSP (s)	1.07(0.99-1.13)	1.04 (1.01-1.13)	1.05 (1.00-1.11)
Stride length SSP (cm)	122.86 (114.75-134.36)	114.85 (96.30-124.39)**	106.09(88.69-120.39)**
Velocity FP (cm/s)	146.99 (139.58-167.63)	126.93 (109.13-142.85)**	123.74 (103.04-137.72)**^#^
Cadence FP (step/min)	129.62 (120.38-136.41)	123.43 (114.61-132.22)	125.84 (114.93-132.32)
Stride time FP (s)	0.93 (0.88-1.00)	0.97 (0.91-1.05)	0.95 (0.91-1.04)
Stride length FP (cm)	137.24 (127.28-150.49)	122.72(108.65-138.50)*	120.43(101.37-129.18)**^#^

**Note: **SSP, self-selected pace, FP, fast pace. For control, there is no ON or OFF state. Same set of clinical data from the control was used to analyze against PD group.

*, *p*< 0.05, **, *p* < 0.01, compared with control group.

^#^, *p* < 0.05, compared with ON State.

**Table 3:** Mean tract-specific fractional anisotropy measurements.

Tract	Controls	PD	F	*p* value
Left Thalamic Radiation	0.490 (0.033)	0.492 (0.037)	0.095	0.759
Right Thalamic Radiation	0.500 (0.032)	0.484 (0.051)	0.972	0.329
Left Corticospinal	0.767 (0.037)	0.764 (0.037)	0.481	0.491
Right Corticospinal	0.769 (0.038)	0.771 (0.038)	0.108	0.743
Left Cingulum Cingulate	0.591 (0.043)	0.575 (0.044)	0.925	0.341
Right Cingulum Cingulate	0.615 (0.047)	0.585 (0.043)	4.157	0.047
Left Cingulum Hippocampus	0.466 (0.059)	0.472 (0.066)	0.081	0.777
Right Cingulum Hippocampus	0.458 (0.062)	0.448 (0.063)	0.182	0.672
Callosum Forceps Major	0.742 (0.037)	0.730 (0.043)	0.614	0.437
Callosum Forceps Minor	0.619 (0.047)	0.619 (0.049)	0.359	0.552
Left IFOF	0.588 (0.035)	0.589 (0.029)	0.680	0.413
Right IFOF	0.596 (0.032)	0.583 (0.032)	1.717	0.196
Left ILF	0.530 (0.029)	0.519 (0.029)	1.117	0.296
Right ILF	0.541 (0.026)	0.531 (0.035)	0.904	0.346
Left SLF	0.586 (0.032)	0.567 (0.037)	2.387	0.129
Right SLF	0.527 (0.041)	0.522 (0.034)	0.032	0.860
Left Uncinate	0.463 (0.049)	0.441 (0.055)	1.547	0.219
Right Uncinate	0.489 (0.073)	0.475 (0.050)	0.063	0.803
Left Arcuate	0.561 (0.033)	0.564 (0.043)	0.118	0.732
Right Arcuate	0.574 (0.037)	0.584 (0.029)	2.424	0.126

**Note: **Mean fractional anisotropy represent the average fractional anisotropy of the 100 nodes for each tract. Data are presented as mean (standard deviation). IFOF, inferior fronto-occipital fasciculus; ILF, inferior longitudinal fasciculus; SLF, superior longitudinal fasciculus.

## Data Availability

The data supporting the findings of this study are available within the article.

## LIST OF ABBREVIATIONS

HCs: Healthy Controls
rCC: Right Cingulum Cingulate
PD: Parkinson's Disease
FOG: Freezing of Gait
DTI: Diffusion Tensor Imaging
PIGD: Postural Instability and Gait Disturbances
FA: Fractional Anisotropy
SN: Substantia nigra
SNpc: Substantia nigra pars compacta
SD: Spherical Deconvolution
ODF: Orientation Distribution Function
MSMT-CSD: Multi-shell Multi-tissue CSD
BBS: Berg Balance Scale
BAI: Beck Anxiety Inventory
MAES: Modified Apathy Evaluation Scale
NFOGQ: New Freezing of Gait Questionnaire
TUG: Timed-Up and Go
SSP: Self-selected Pace
FP: Fast pace
FWE: Family-wise Error
TFCE: Threshold-free Cluster Enhancement

## STANDARDS OF REPORTING

STROBE guidelines were followed.
